# No association between cortical dopamine D2 receptor availability and cognition in antipsychotic-naive first-episode psychosis

**DOI:** 10.1038/s41537-021-00176-x

**Published:** 2021-09-21

**Authors:** Maria Lee, Helena Fatouros-Bergman, Pontus Plavén-Sigray, Pauliina Ikonen Victorsson, Carl M. Sellgren, Sophie Erhardt, Lena Flyckt, Lars Farde, Simon Cervenka

**Affiliations:** 1 Centre for Psychiatry Research, Department of Clinical Neuroscience, Karolinska Institutet, & Stockholm Health Care Services, Region Stockholm, Stockholm, Sweden; 2 Department Neurology and Neurobiology Research Unit, Copenhagen University Hospital, Rigshospitalet, Denmark; 3 Department of Physiology and Pharmacology, Karolinska Institutet, SE- 171 77 Stockholm, Sweden; 4 Department of Neuroscience, Psychiatry, Uppsala University, Uppsala, Sweden

**Keywords:** Schizophrenia, Working memory, Molecular neuroscience

## Abstract

Cognitive impairment is an important predictor of disability in schizophrenia. Dopamine neurotransmission in cortical brain regions has been suggested to be of importance for higher-order cognitive processes. The aim of this study was to examine the relationship between extrastriatal dopamine D2-R availability and cognitive function, using positron emission tomography and the high-affinity D2-R radioligand [^11^C]FLB 457, in an antipsychotic-naive sample of 18 first-episode psychosis patients and 16 control subjects. We observed no significant associations between D2-R binding in the dorsolateral prefrontal cortex or hippocampus (*β* = 0.013–0.074, partial *r* = −0.037–0.273, *p* = 0.131–0.841). Instead, using Bayesian statistics, we found moderate support for the null hypothesis of no relationship (BF_H0:H1_ = 3.3–8.2). Theoretically, our findings may suggest a lack of detrimental effects of D2-R antagonist drugs on cognition in schizophrenia patients, in line with clinical observations.

## Introduction

Diagnostic criteria for schizophrenia include positive symptoms (delusions, hallucinations, disorganized speech, and behavior) and negative symptoms (loss of motivation, blunted affect, etc.). Beyond these symptoms, a majority of patients also have a cognitive impairment, with global functioning significantly below that of healthy controls (HCs)^1^. The cognitive domains that are most severely affected include verbal learning and memory, processing speed, working memory, and executive function, and the level of dysfunction seems to be similar in medicated^2–4^ and antipsychotic drug-naive patients^5^. In addition, it has been hypothesized that the pathology underlying cognitive impairment also contributes to positive symptoms^6^ and cognitive status has been shown to predict functional outcomes better than any other symptom dimension^2,7^.

Molecular imaging studies using positron emission tomography (PET) and single-photon emission computed tomographic (SPECT) have provided evidence of the abnormal function of the dopamine system in schizophrenia; primarily in the form of elevations in presynaptic dopamine synthesis capacity and amphetamine-induced dopamine release in striatal regions^8^. With regard to receptor subtypes, pharmacological and genetic evidence provides indirect support for an involvement of the dopamine D2 receptor (D2-R) in the pathophysiology of schizophrenia^9–11^. PET studies have shown a slight increase of this receptor subtype in schizophrenia in striatum^8^, and in parallel striatal D2-R has in several studies been reported to be associated with measures of processing speed and executive function in healthy subjects^12–14^ as well as in drug-free patients with schizophrenia^15^.

A wealth of literature has established that the frontal cortex and hippocampus are key regions for higher-order cognition in humans. In patients with schizophrenia, altered activation in these regions has been linked to cognitive performance, using functional resonance imaging^16–18^. Given the role of D2-R in cognition, surprisingly few studies have investigated the relationship between D2-R availability and cognition in extrastriatal regions. Four studies have so far investigated this relationship in patients with schizophrenia revealing no clear pattern of associations^19–22^. Moreover, the observed correlations in these studies differ from those observed in HC; either showing associations in the opposite direction or a lack of a significant relationship^23–26^ (see Supplementary Table 1).

The purpose of this study was to examine the relationship between D2 receptor availability and cognitive function in an antipsychotic drug-naive sample of first-episode psychosis (FEP) patients and HC subjects. Based on the existing literature, we selected specific cognitive domains and brain regions a priori to preserve sufficient statistical power, and we utilized both frequentist and Bayesian statistics to gain more information about potential associations.

## Results

Data on demographics are presented in Table 1. FEP and HC did not differ significantly in terms of age, gender, body mass index, nicotine use, or years of education.

**Table 1:** Demographic and clinical data, mean (standard deviation).

	FEP *N* = 18	HC *N* = 16	Fisher’s exact test	*t* value	*df*	*p* value
Gender (male/female)	11/7	10/6	OR = 1.1			>0.99
Age	28.9 (6.3)	29.3 (6.2)		0.28	32.98	0.782
BMI	23.5 (2.5) *N* = 17	24.7 (3.2) *N* = 15		−1.20	28.87	0.240
Education in years	15.2 (3.3) *N* = 17	15.0 (2.2)		0.06	28.05	0.952
Nicotine user (yes/no)	6/12	2/13 *N* = 15	OR = 2.9			0.257
Duration of illness (months)	12.7 (19.9)	-				
*PANSS*		-				
Positive	17.4 (4.2)					
Negative	14.6 (6.3)					
General	34.0 (9.8)					
Total	66.1 (17.7)					
*Level of functioning*		-				
CGI	4.5 (1.0)					
GAF	41.9 (13.5)					

*BMI* body mass index, *OR* Odds ratio, *FEP* first-episode psychosis.

The regions of interest (ROI) for the confirmatory analyses were the dorsolateral prefrontal cortex (DLPFC) with a mean [^11^C]FLB 457 binding potential (BP_ND_) of 0.80 (SD 0.23) for patients and 0.83 (SD 0.32) for controls; as well as the hippocampus with a mean BP_ND_ of 1.27 (SD 0.24) for patients and 1.17 (SD 0.20) for controls. In Table 2, the Measurement and Treatment Research to Improve Cognition in Schizophrenia (MATRICS) Consensus Cognitive Battery (MCCB) cognitive tests and domains, used in the confirmatory analyses are presented. The values listed are raw scores to allow for comparison with other samples, however, both cognition and BP_ND_ values were age-corrected in our analysis. See the Methods section for more information. Statistically significant mean differences were observed for four out of seven cognitive domains, when comparing raw scores; Speed of Processing, Visual Memory, Attention, and Neurocognitive Composite (see Supplementary Table 2).

**Table 2:** Raw scores of MCCB cognitive domains (**in bold**) and their respective cognitive tests, mean (standard deviation).

Cognitive domain	FEP	HC	*t* value	*df*	*p* value
Cognitive test(s)	*N* = 18	*N* = 16			
**Speed of processing**	67.15 (8.2)	74.77 (4.5)	−3.42	27.13	0.002
Trail making test A	129.5 (12.4)	135.38 (6.2)	−1.78	25.57	0.087
BACS symbol coding	50.89 (13.7)	61.44 (8.5)	−2.72	28.77	0.011
Category fluency, animal naming	21.06 (5.8)	27.5 (5.1)	−3.45	32.00	0.002
**Working memory**	16.00 (3.0)	16.47 (2.2)	−0.52	30.99	0.606
WMS-III spatial span	17.22 (3.4)	17.69 (2.5)	−0.46	31.05	0.648
WAIS-III letter-number sequencing	14.78 (3.6)	15.25 (2.8)	−0.42	31.31	0.671
**Verbal memory**^1^	26.83 (4.7)	28.94 (3.4)	−1.50	30.62	0.143
Hopkins verbal learning test–revised					

^1^The verbal memory domain consists of only one test; therefore, raw scores for the domain and the cognitive test are the same.

### Confirmatory findings

**Fig. 1:**
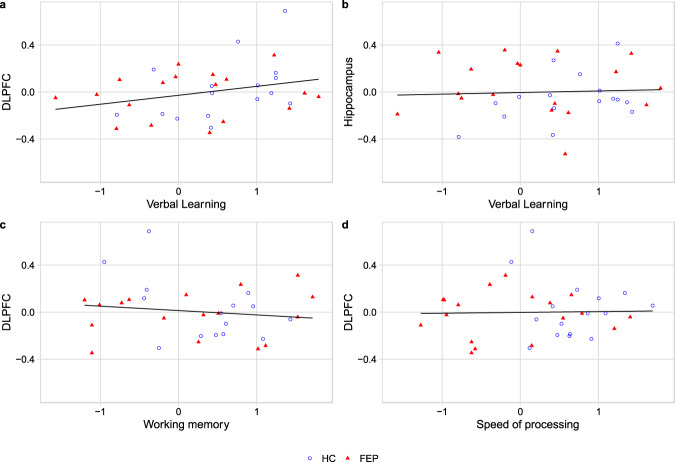
Relationship between [^11^C]FLB 457 BP_ND_ values and age-corrected cognitive test scores for the confirmatory analyses. Blue circles indicate HC, red triangles indicate FEP. The gray area indicates a 95% confidence interval for the regression line. **a** BP_ND_ in DLPFC and verbal learning, **b** BP_ND_ in the hippocampus and verbal learning, **c** BP_ND_ in DLPFC and working memory, **d** BP_ND_ in DLPFC and speed of processing.

Findings from the regression models are presented in Tables 3 and 4. The main effect of cognition on BP_ND_ was not found to be statistically significant for any of the tests and regions investigated (*β* = 0.013–0.074, *p* = 0.131–0.841, partial *r* = −0.037–0.273) (Fig. 1). None of the interaction effects were statistically significant.

**Table 3:** Confirmatory analyses, the main effect of cognition.

	*β* (95% CI)	Partial *r*	Df	*p* value	BF_H0:H1_
Verbal learning and D2-R in DLPFC	0.074 (−0.023–0.171)	0.273	30	0.131	3.3
Verbal learning and D2-R in hippocampus	0.031 (−0.069–0.132)	0.115	30	0.529	8.2
Working memory and D2-R in DLPFC	−0.039 (−0.123–0.044)	−0.174	30	0.341	7.6
Speed of processing and D2-R in DLPFC	−0.013 (−0.140–0.115)	−0.037	30	0.841	7.7

**Table 4:** Confirmatory analyses with an added interaction effect.

	*β* (95% CI) interaction effect	Df	*p* value	Partial *r* FEP	Df	*p* value FEP	Partial *r* HC	Df	*p* value HC
Verbal learning D2-R DLPFC	−0.143 (−0.352–0.066)	29	0.173	0.134	15	0.608	0.443	13	0.098
Verbal learning D2-R hippocampus	−0.137 (−0.354–0.080)	29	0.207	−0.034	15	0.896	0.419	13	0.120
Working memory D2-R DLPFC	0.134 (−0.039–0.306)	29	0.124	0.060	15	0.819	−0.402	13	0.138
Speed of processing D2-R DLPFC	0.097 (−0.203–0.397)	29	0.513	0.020	15	0.938	−0.114	13	0.685

BF in favor of H0 relative to H1 were all above 3 (BF_H0:H1_ 3.3–8.2, see Table 3), indicating moderate evidence in favor of the null hypothesis of no relationship between cognitive function and D2-R availability^27^. The main confirmatory analyses were re-run without the three FEP individuals with a diagnosis of delusional disorder (Supplementary Tables 3, 4), yielding similar results.

Gender effects on cognition have been observed in the general population using the MCCB^28,29^, as well as in samples of patients with schizophrenia^30^. We did not set out to test the potential impact of gender in this study, given our small sample size. Post hoc, we added gender as a covariate to our model, but this did not change the results (see Supplementary Table 5).

### Exploratory analyses

For descriptive statistics on the exploratory cognitive domains, see Supplementary Table 2. In the analyses of the main effect of cognition, attention was positively correlated with D2-R availability in several ROIs (hippocampus: *r* = 0.521, temporal cortex (TC): *r* = 0.474, anterior cingulate cortex (ACC): *r* = 0.326, thalamus: *r* = 0.300). All other associations between regional BP_ND_ and cognitive domains showed small partial correlation values, below 0.3 (see Supplementary Table 6). When including the effect of FEP/HC status, an interaction effect was observed for the relationship between visual learning and BP_ND_ in the hippocampus. After examining the partial correlations for the groups separately, this relationship was driven by the HC who showed a medium-sized positive correlation (*r* = 0.613), whereas FEP shows a negligible negative correlation (*r* = −0.184) (Supplementary Tables 7, 8).

### Longitudinal analyses

Partial correlations between [^11^C]FLB 457 BP_ND_ and cognition at 1.5-year follow-up were explored. Associations of a moderate magnitude (partial *r* = 0.5) were found for the relationship between D2-R availability in the hippocampus and the cognitive domain attention (see Supplementary Table 9). Exploratory analyses between the baseline BP_ND_ levels and change in cognitive test results from baseline to follow-up were also performed, see Supplementary Table 10.

## Discussion

In this study, we investigated the relationship between regional dopamine D2 receptor binding and cognition in a well-defined cohort of drug-naive FEP and matched HC. We used high-resolution PET, a radioligand with very high affinity, well suited to quantify D2-R availability in low-density extrastriatal brain regions^31^, and a battery of cognitive tests specifically developed to be sensitive to cognitive impairment in schizophrenia. Contrary to our hypotheses, no significant associations between D2-R availability in pre-defined regions and cognitive function were observed. Instead, we obtained moderate support in favor of the null hypothesis of no relationship between D2-R and cognition, relative to the alternative hypothesis. This suggests a lack of a sizeable, and thereby clinically relevant, the relationship between D2-R availability in the frontal cortex and the hippocampus and working memory, speed of processing, or verbal learning in FEP and HC.

This study was not designed to test the effects of antipsychotic drugs on cognition. However, the absence of an association between extrastriatal D2-R availability and cognition may be viewed in light of the fact that all currently available antipsychotic drugs act by occupying this receptor subtype. Whereas experimental studies in HC have demonstrated a negative effect on cognition by antipsychotic drugs^32^, clinical studies on patients with psychotic disorders have produced more mixed results^33^. Although some studies report negative effects^34–36^ others report positive^37,38^ or no effect on cognition^39^. Moreover, meta-analyses of cognitive function in drug-naive FEP show the same level of impairment as in patients who have been treated with D2-R blocking drugs^4,5^. Theoretically, the lack of relationship between D2-R and cognition in the present study is in line with the view that D2-R blocking drugs may not worsen cognitive function in patients with schizophrenia.

Previous studies have reported associations between D2-R availability and cognition that differ between patients with schizophrenia and HC. In some cases, significant correlations have been reported in one group but not the other^20,21^, and in other cases, the direction of the relationship has been reversed between groups^19,21^. The idea of an altered impact of dopamine on cognitive function in schizophrenia motivated us to explore an interaction effect of FEP/HC status on the relationship between cognitive functioning and D2-R availability. However, we found no support in our data for this hypothesis.

In the exploratory analysis, a sizeable positive correlation was observed between attention and D2-R in the hippocampus, TC, ACC, and thalamus. Attention has been considered to be the foundation for other higher-order cognitive abilities known to be impaired in schizophrenia, as well as related to positive symptoms of the disease^2,40^. Moreover, the prefrontal cortex and the ACC have been associated with measures of attention in the previous lesion and imaging studies^41^. This potential association warrants further investigation in other independent samples of patients with schizophrenia and controls.

The existing literature on the association between D2 receptor availability and cognitive performance in patients with schizophrenia has produced mixed results (see Supplementary Table 1). Sliftstein et al.^20^ found no relationship between working memory and basal D2-R availability (BP_ND_) in the DLPFC as determined using [^11^C]FLB 457 PET. Using the same radioligand, Rao et al.^22^ tentatively observed a negative correlation between D2-R availability in the DLPFC and language/speed of processing measures in an exploratory analysis. Another study observed a positive linear correlation between D2-R binding in the frontal cortex and set-shifting in antipsychotic-naive FEP, using the SPECT radiotracer [^123^I]epidepride^19^. Moreover, quadratic correlations between D2-R and planning efficiency, planning latency, and selective attention were observed. Finally, ^18^F-fallypride has been used to demonstrate negative correlations between D2-R in several brain regions and verbal learning as well as executive function in patients with schizophrenia^21^. In summary, previous imaging studies have utilized different techniques and radioligands, while investigating varying cortical regions and cognitive outcome measures, limiting the conclusions that can be drawn. Moreover, in these studies, it may be argued that there was an insufficient correction for multiple comparisons. We sought to improve on this in the present study by defining our analyses a priori and correcting for multiple comparisons. An additional strength is that we utilized Bayesian methods to gain more information from a non-significant result^42^, by quantifying the relative support for the null hypothesis against a skeptical but plausible alternative hypothesis.

As with previous studies in the field, the major limitation of our study was the sample size. The study was powered to detect a medium effect size, and in order to detect more subtle associations, larger samples are needed. Notably, we were not able to demonstrate significant differences in performance between FEP and HC on several cognitive measures previously shown to be impaired in patients (working memory, verbal learning, and executive function). PET examination and cognitive testing were performed at different times, and two FEP were no longer drug-naive at the time of cognitive testing. Cognitive testing of patients in the initial phase of their first psychotic episode introduces potential bias; psychotic symptoms, sleep deprivation, and/or stress likely introduce some noise in our cognitive outcome measures. This weakness has to be weighed against the advantages of obtaining [^11^C]FLB 457 PET values at the first onset of the disease, before exposure to the confounding effects of antipsychotic medication. A related concern is a potential for selection bias with our study design. Recruiting drug-naive FEP patients who are able to consent and take part in a PET study is challenging and might result in patients who have milder symptoms (able to post-pone drug treatment) and are more positively inclined towards research in general (potentially related to educational level). The fact that substance use was an exclusion criterion also limits the generalizability of our findings to the general population of patients with schizophrenia. Given these inherent challenges, we still conclude that our sample is not atypical compared with other molecular imaging studies using drug-naive or drug-free FEP in terms of age, duration of untreated psychosis, or symptom burden^19,21,22^.

Our main finding is moderate support for no relationship between D2-R and cognition in a sample of FEP, as quantified using Bayes Factor. This is in line with clinical data suggesting a lack of significant negative effects of antipsychotic drugs on cognitive function in patients with schizophrenia. More work is needed to understand how aberrations in dopamine function may impact different symptom domains in schizophrenia, preferably using larger samples and collaborations between imaging centers to gain more conclusive evidence^43^.

## Methods

### Participants

The study was approved by the Regional Ethics Committee in Stockholm (diary number: 2010/879-31-1) as well as the Radiation Safety Committee of the Karolinska University Hospital. Subjects were included after providing written informed consent.

Twenty antipsychotic-naive, FEP patients were included from three psychiatric clinics in Stockholm, as part of the Karolinska Schizophrenia Project (KaSP). One participant was later excluded owing to brain abnormalities demonstrated by magnetic resonance imaging (MRI) and age (65 years at scan) and one participant was excluded due to missing MRI, making the final number of FEP 18 (11 male, seven female, mean age 28.9 years (SD 6.3)). At the time of PET investigation, all FEP were naive to antipsychotic treatment and met the diagnostic criteria for schizophrenia (*N* = 6), schizophreniform disorder (*N* = 5), psychotic disorder NOS (*N* = 4), or delusional disorder (*N* = 3) according to DSM-IV. Exclusion criteria were neurologic or severe somatic illness and current use or history of abuse of illegal drugs (including cannabis). The Alcohol Use Disorders Identification Test (AUDIT) and Drug Use Disorders Identification Test (DUDIT) were used to assess the use of illegal drugs and alcohol abuse, along with a urine drug screen test prior to the PET scan.

Sixteen HC subjects (10 male, 6 female, mean age 29.3 years (SD 6.2)) were recruited by advertisement. Subjects were healthy according to medical history, clinical examination, routine laboratory blood test as well as a brain MRI examination. The Mini-International Neuropsychiatric Interview (MINI) was used to exclude previous or current psychiatric illness. Further exclusion criteria were previous or current use of illegal drugs (assessed using AUDIT and DUDIT, and urine toxicology prior to PET) and first-degree relatives with psychotic illness. See Table 1 for demographic and clinical data.

Participants underwent PET examination and cognitive testing on different days, with a mean of 5.9 days (SD 5.1) between examinations. At the time of cognitive testing, two FEP had been prescribed first-generation antipsychotics, with 3 and 6 days of total exposure respectively.

PET data on the participants of the present study is partly overlapping with a multimodal imaging study where PET and the high-affinity radioligand [^11^C]FLB 457, as well as diffusion-weighted imaging, was used to examine group differences in thalamic D2-R and connectivity, the results of which will be reported elsewhere.

### Cognitive tests

Cognitive function was assessed using the MCCB^28,44^ as well as the Wisconsin Card Sorting Test (WCST)^45^. The MCCB was developed specifically to study cognitive domains known to be affected in schizophrenia and evaluates speed of processing, attention/vigilance, working memory, verbal learning, visual learning, reasoning/problem solving, and social cognition (See Supplementary Table 2 for a detailed description of the cognitive tests that make up each domain). WCST is a measure of executive function, specifically set-shifting. All FEP completed the MCCB, and all but two performed the WCST. All HC completed all cognitive tasks. The main outcome measures specified in the MCCB manual for the different cognitive domains were used, as well as percentage errors for the WCST.

A subgroup of FEP (*N* = 12, out of which 10 had a psychotic disorder) completed cognitive testing at 1.5-year follow-up (mean 590.92 days (SD = 66.07), see Supplementary Table 11 for additional information), using the same cognitive tests as described above. For this subgroup repeated measures of cognition were analyzed in the exploratory section. All other analyses involved measurements taken from two distinct samples described above; FEP and HC.

### Image data acquisition and analysis

Structural MRI was obtained at Karolinska University Hospital, Solna, using a 3-T General Electric Discovery MR750 system (GE, Milwaukee, WI, USA). T1-weighted images were used for the ROI delineation. Study participants were then examined at the Karolinska Institutet PET center, using a high-resolution research tomograph (Siemens Molecular Imaging, Knoxville, TN, USA). A transmission scan with a 137Cs source was performed prior to the emission scan to correct for attenuation. [^11^C]FLB 457 was prepared using in-target produced [^11^C] methane^46^ and injected as a rapid bolus into the antecubital vein. Mean injected radioactivity was 430.2 (SD 45.0) MBq, mean molar activity was 392.2 (SD 205.4) Gbq/micromole, and mean injected mass was 0.54 (SD 0.31) mg. All emission scans were 90 min long.

PET images were reconstructed into 3 × 1min, 4 × 2 min, and 12 × 6 min subsequent frames using ordered subset expectation maximization. Head movement was further corrected by frame-to-frame realignment^47^. T1 images were co-registered to summation PET images using SPM5 (Wellcome Trust Centre for Neuroimaging, London, UK) in Matlab 2014 (The Mathworks, Natick, MA, USA). All ROIs were then back-transformed using MRI-PET coregistration parameters and applied to the PET data. From this, regional time-activity curves were extracted and used in kinetic modeling.

Two FEP went out of the system during frame 14 and frames 7–9, respectively, owing to experiencing discomfort. For both individuals, a new transmission scan was then obtained for subsequent frames and the missing frames were excluded from the ensuing quantification.

The Logan Graphical Analysis^48^ with cerebellum as reference region was used to obtain BP_ND_ values for all ROIs. All quantification was performed using the “kinfitr” package in R^49^.

### Analytic design and statistics

Considering only a few previous studies have investigated D2-R in relation to cognitive function in FEP, we chose to examine our data in two ways; a confirmatory part and an exploratory part. This statistical study design was pre-registered and is publicly available at https://aspredicted.org/cf82g.pdf.

### Confirmatory analyses

The confirmatory analyses were hypothesis-driven, examining associations between cognitive domains that are of particular importance in schizophrenia and brain regions thought to be involved in those cognitive processes. The cognitive domains chosen were verbal learning, working memory, and processing speed, as these domains have been identified as the most impaired in a meta-analysis of antipsychotic drug-naive FEP^5^. ROIs were DLPFC (for verbal learning, working memory, and speed of processing) as dysfunction in this region has been associated with schizophrenia pathology in general and with several forms of cognitive impairment;^16,18^ and the hippocampus (for verbal learning) given its specific importance in learning and memory for patients^17^.

First, we analyzed the relationship between D2-R availability and cognition in the whole sample. Thereafter, we repeated the analysis with an added interaction effect for FEP/HC status (as previous studies have pointed towards differential relationships between cognition and dopamine for FEP and HC, respectively (see Supplementary Table 1)). At last, we applied a Bayesian analysis in the confirmatory section, by calculating Bayes Factors, to quantify how much the data supports the alternative hypothesis over the null hypothesis and vice versa^42,50^.

### Age-correction of cognitive test scores

Association between age and performance on cognitive tests is well-documented^41^, motivating the use of age-corrected cognitive test scores. Participants of this study were part of a larger cohort of FEP (*N* = 83) and HC (*N* = 59), who were tested with the same test battery. Inclusion and exclusion criteria were identical for this cohort as for the PET participants, with the exception that many FEP patients (*N* = 43) had been exposed to antipsychotic medication at the time of cognitive testing. For additional demographics, see Supplementary Tables 12, 13. As no Swedish norming of the MCCB exists, cognitive test scores were age-corrected using linear regression (raw cognitive test score ~ age) using the larger cohort of FEP and HC.

### Power analysis and correction for multiple comparisons

We performed a power calculation using the lower bound of reported correlations from previous literature and the aim of being able to detect a correlation of moderate magnitude (considered to be an *r* = 0.5) in our sample. Cognitive results and BP_ND_ values in our selected ROIs were intercorrelated (cognitive tests *r* = 0.27–0.50; ROIs *r* = 0.35), thus a Bonferroni correction for multiple comparisons was deemed too conservative. Instead, the alpha level was corrected by estimating the effective number of tests (Meff), an analytical approach using the correlations among the variables being tested while maintaining a consistent total family-wise error rate of 5%^51^. The ensuing significance threshold for the confirmatory tests was set to 0.027.

### Linear regression models

From the power analysis, based on the corrected alpha, fixed sample size, and a beta of 0.2, we chose to perform four confirmatory linear regression models with [^11^C]FLB 457 BP_ND_ in ROIs as the dependent variable and (age-corrected) cognitive test scores as the independent variable. FEP/HC status and age were added as covariates. The following two-sided hypotheses were tested:

1. Verbal learning is correlated with D2-R BP_ND_ in the DLPFC.
2. Verbal learning is correlated with D2-R BP_ND_ in the hippocampus.
3. Working memory is correlated with D2-R BP_ND_ in the DLPFC.
4. Speed of processing is correlated with D2-R BP_ND_ in the DLPFC.

The significance threshold was kept on the same level for a secondary regression step, adding an interaction term for FEP/HC status, meaning that a marginally elevated overall alpha level was accepted. Pearson partial correlations were also calculated for the regression models.

For the Bayesian analysis, the null hypothesis (H0: there is no association between BP_ND_ and cognitive test scores) was defined so that the beta-coefficient denoting the BP_ND_-cognition relationship was equal to zero. The alternative hypothesis (H1: there is an association between BP_ND_ and cognitive test scores) was defined so that the beta-coefficient was distributed according to a normal distribution, centered around zero with an SD of 0.5, meaning that BP_ND_ and cognitive test scores are related in some way, and gives higher prior plausibility to smaller relationships relative to larger relationships.

### Exploratory analyses

The exploratory section utilized additional extrastriatal ROIs (thalamus, ACC, and TC) and cognitive domains (attention, visual learning, executive function, and neurocognitive composite) to investigate any potential relationship between cognition and D2-R. Associations were investigated using linear regression, with and without interaction effect, as well as Pearson partial correlation. Results were not corrected for multiple comparisons and should be viewed as hypothesis-generating and not hypothesis testing. For this reason, *p* values are solely reported as a continuous measure of indirect evidence against the null hypothesis^52^, and not interpreted in a dichotomous manner. Furthermore, potential relationships between D2-R levels at baseline and cognitive performance at 1.5-year follow-up, (as well as the relationship between D2-R levels at baseline and change in the level of cognitive function from baseline to follow-up) were explored for the cognitive tests and brain regions mentioned above.

All statistical analyses were performed using the statistical software R version 3.6.2^53^.

### Reporting summary

Further information on research design is available in the Nature Research Reporting Summary linked to this article.

## Supplementary information

Supplementary Information

Reporting Summary

## Data Availability

Owing to institutional restrictions, the data cannot be shared openly but can instead be made available upon request on a case-by-case basis as allowed by the legislation and ethical permits. Requests for access can be made to the Karolinska Institutet’s Research Data Office at rdo@ki.se.

## Code availability

The code for reproducing the analyses and figures in this article can be found at https://github.com/MariaLeeR/D2_cognition_FEP.
