# Supplementary material for: No association between cortical dopamine D2 receptor availability and cognition in antipsychotic-naive first-episode psychosis
Source: NPJ Schizophr. 2021 Sep 21;7:46. doi: 10.1038/s41537-021-00176-x (PMC8455597; doi:10.1038/s41537-021-00176-x)
Supplement: Supplementary file 1 — Supplementary Information [file 41537_2021_176_MOESM1_ESM.pdf]

Supplementary material to the article “No association between cortical dopamine D2-receptor availability and cognition in anti-psychotic naïve first episode psychosis”

Supplementary Table 1:
Previous studies of cognitive ability and its relationship to dopamine D2-R in healthy controls and in schizophrenia patients. All results listed reflect the relationship between better performance and higher BP_{ND} levels, if error measures were used the correlation has been reversed

Supplementary Table 1

	Population	Ligand	Region	Cognitive function	Relation to D2-R levels
**Rao, 2018**	13 SCZ (5 anti-psychotic naïve/8 drug free) + 14 HC	$[^{11}C]$FLB 457	DLPFC<br><br>DLPFC	Verbal learning and memory<br>Language/speed of processing	SCZ: 0<br>HC: -<br>SCZ: -<br>HC: 0
**Vyas, 2018**	25 SCZ (20 anti-psychotic naïve/5 drug free) + 19 HC	^{18}F-fallypride	DLPFC, PFC, hippocampus, thalamus<br><br>Dorsal striatum, thalamus, limbic	Verbal learning and memory<br><br>Executive function	SCZ: -<br>HC: 0<br><br>SCZ: -<br>HC: +
**Slifstein, 2015**	20 SCZ (6 anti-psychotic naïve/14 drug free) + 21 HC	$[^{11}C]$FLB 457	DLPFC	Working memory	SCZ: 0<br>HC: +
**Fagerlund, 2013**	24 FEP anti-psychotic naïve +<br>20 HC	SPECT $[^{123}I]$epidepride	Frontal cortex	Executive function (set-shifting)<br>Executive function (planning)<br>Attention	SCZ: +<br>HC: ∪<br>SCZ: ∩<br>HC: ∩<br>SCZ: ∩<br>HC: ∪
**Lumme, 2010**	40 HC	$[^{11}C]$FLB 457	ACC, PFC, hippocampus amygdala etc.	Verbal memory	HC: 0
**Takahashi 2008,**	23 HC	$[^{11}C]$FLB 457	Hippocampus<br><br>PFC	Visual memory<br><br>Executive function	HC: +<br><br>HC: +
**Takahashi, 2007**	25 HC	$[^{11}C]$FLB 457	Hippocampus<br><br>Hippocampus	Memory<br><br>Executive function	HC: +<br><br>HC: +
**Lumme, 2007**	32 HC	$[^{11}C]$FLB 457	Right ACC	Executive function	HC: -

+ = positive linear correlation, - = negative linear correlation, 0 = no significant correlation, ∪ = U-shaped quadratic correlation, ∩ = inverted U-shaped quadratic correlation. All results reflect the relationship between better performance and higher BP_{ND} levels, if error measures were used the correlation has been reversed. HC = healthy control subjects, SCZ = schizophrenia patients, FEP = first episode psychosis patients.

Supplementary Table 2 lists all the cognitive domains of the MCCB as well as the test of Executive Function (WCST), and the raw values of these for patients and controls in the present study. P-values have not been corrected for multiple comparisons.

Supplementary Table 2. Cognitive domains and tests for exploratory analyses (raw scores, mean (standard deviation))

**Cognitive domain**<br>Cognitive test(s)	FEP<br>N = 18	HC<br>N = 16	t-value	df	p-value
**Speed of processing**<br>Trail Making Test A<br>BACS Symbol Coding<br>Category Fluency, Animal naming	67.15 (8.2)	74.77 (4.5)	-3.42	27.13	0.002*
**Working memory**	16.00 (3.0)	16.47 (2.2)	-0.52	30.99	0.606
WMS-III Spatial Span					
WAIS-III Letter number sequencing					
**Verbal memory**	26.83 (4.7)	28.94 (3.4)	-1.50	30.62	0.143
Hopkins Verbal Learning Test - Revised					
**Visual memory**	24.11 (6.8)	30.06 (2.7)	-3.40	22.77	0.003*
Brief Visuospatial Memory Test-Revised					
**Attention**	2.39 (0.6)	2.82 (0.5)	-2.13	31.66	0.041*
Continuous Performance Test – Identical Pairs Version					
**Neurocognitive composite**	155.38 (19.6)	174.77 (10.6)	-3.64	26.76	0.001*
All tests above + NAB Mazes					
**Executive function**	21.94 (11.1)	19.63 (14.4)	0.51	28.15	0.616
Wisconsin Card Sorting Test (% Errors)	N=16				

Post-hoc analyses.

Main analysis repeated without FEP who had a diagnosis of delusional disorder (N = 3).
FEP N = 15, HC N = 16

Supplementary Table 3. Subgroup analysis, main effect of cognition

	β (95% CI)	Partial *r*	Df	*p*-value
**Verbal learning and D2R in DLPFC**	0.096 (-0.008 – 0.200)	0.343	27	0.068
**Verbal learning and D2R in Hippocampus**	0.047 (-0.064 – 0.157)	0.164	27	0.395
**Working memory and D2R in DLPFC**	-0.058 (-0.149 – 0.033)	-0.244	27	0.203
**Speed of processing and D2R in DLPFC**	-0.018 (-0.158 – 0.123)	-0.050	27	0.800

Supplementary Table 4. Subgroup analyses, with added interaction effect

	β (95% CI) interaction effect	Df	*p*-value	Partial *r* FEP	Df	*p*-value FEP	Partial *r* HC	Df	*p*-value HC
**Verbal learning D2R DLPFC**	-0.118 (-0.337 – 0.100)	26	0.277	0.249	12	0.391	0.443	13	0.098
**Verbal learning D2R Hippocampus**	-0.124 (-0.356 – 0.108)	26	0.283	0.018	12	0.951	0.419	13	0.120
**Working memory D2R DLPFC**	0.114 (-0.070 – 0.299)	26	0.214	-0.054	12	0.854	-0.402	13	0.138
**Speed of processing D2R DLPFC**	0.086 (-0.227 – 0.400)	26	0.576	-0.003	12	0.990	-0.114	13	0.685

Main analysis repeated with gender as a covariate.

Supplementary Table 5.

	Original results (age + patient/HC status)		Results with gender as added covariat	
	β (95% CI)	*p*-value	β (95% CI)	*p*-value
**Verbal learning and D2R in DLPFC**	0.074 (-0.023 – 0.171)	0.131	0.076 (-0.024 – 0.175)	0.130
**Verbal learning and D2R in Hippocampus**	0.031 (-0.069 – 0.132)	0.529	0.034 (-0.068 – 0.136)	0.506
**Working memory and D2R in DLPFC**	-0.039 (-0.123 – 0.044)	0.341	-0.039 (-0.125 – 0.047)	0.365
**Speed of processing and D2R in DLPFC**	-0.013 (-0.140 – 0.115)	0.841	-0.014 (-0.144 – 0.116)	0.826

The following tables (Supplementary Tables 6-10) present all the exploratory analyses conducted.

Supplementary Table 6. Exploratory analyses of D2-R levels and main effect of cognition

	Region of interest														
	**DLPFC**			**Hippocampus**			**Thalamus**			**ACC**			**Temporal cortex**		
**Cognitive domain**	β (95% CI)	Partial *r*	p-value	β (95% CI)	Partial *r*	p-value	β (95% CI)	Partial *r*	p-value	β (95% CI)	Partial *r*	p-value	β (95% CI)	Partial *r*	p-value
**Verbal learning**	-	-	-	-	-	-	-0.072 (-0.333 – 0.189)	-0.102	0.578	0.029 (-0.073 – 0.131)	0.107	0.560	0.120 (-0.024 – 0.263)	0.297	0.098
**Working memory**	-	-	-	0.031 (-0.053 – 0.115)	0.136	0.459	0.038 (-0.182 – 0.258)	0.065	0.725	0.005 (-0.081 – 0.091)	0.023	0.903	-0.019 (-0.145 – 0.107)	-0.057	0.756
**Speed of processing**	-	-	-	0.040 (-0.087 – 0.167)	0.116	0.527	0.053 (-0.278 – 0.384)	0.060	0.745	0.030 (-0.099 – 0.159)	0.087	0.637	0.000 (-0.190 – 0.190)	0.000	0.999
**Attention**	0.068 (-0.029 – 0.164)	0.253	0.162	0.140 (0.054 – 0.225)	0.521	0.002	0.208 (-0.039 – 0.456)	0.300	0.095	0.089 (-0.007 – 0.184)	0.326	0.068	0.188 (0.058 – 0.319)	0.474	0.006
**Visual learning**	0.029 (-0.081 – 0.139)	0.097	0.599	-0.005 (-0.115 – 0.106)	-0.015	0.935	-0.020 (-0.306 – 0.267)	-0.026	0.889	0.051 (-0.059 – 0.161)	0.170	0.353	-0.004 (-0.168 – 0.160)	-0.009	0.963
**Executive function N = 32**	0.060 (-0.039 – 0.159)	0.229	0.224	-0.005 (-0.106 – 0.095)	-0.021	0.913	0.121 (-0.141 – 0.383)	0.176	0.352	0.056 (-0.046 – 0.158)	0.208	0.269	0.096 (-0.051 – 0.243)	0.246	0.191
**General cognitive ability**	0.013 (-0.112 – 0.138)	0.039	0.830	0.037 (-0.087 – 0.161)	0.110	0.551	0.000 (-0.324 – 0.324)	0.000	0.998	0.043 (-0.082 – 0.169)	0.128	0.486	0.028 (-0.157 – 0.213)	0.057	0.759

Supplementary Table 7. Exploratory regression analyses of D2-R levels and cognition with interaction effect

	**Region of interest**									
	**DLPFC**		**Hippocampus**		**Thalamus**		**ACC**		**Temporal cortex**	
	Interaction		Interaction		Interaction		Interaction		Interaction	
**Cognitive domain**	β (95% CI)	p-value	β (95% CI)	p-value	β (95% CI)	p-value	β (95% CI)	p-value	β (95% CI)	p-value
**Verbal learning**	-	-	-	-	-0.419 (-0.978 – 0.139)	0.136	-0.153 (-0.372 – 0.066)	0.164	-0.124 (-0.438 – 0.191)	0.428
**Working memory**	-	-	0.028 (-0.154 – 0.209)	0.759	0.120 (-0.354 – 0.594)	0.609	0.119 (-0.061 – 0.300)	0.187	0.097 (-0.173 – 0.367)	0.468
**Speed of processing**	-	-	-0.109 (-0.409 – 0.190)	0.461	0.102 (-0.687 – 0.890)	0.794	0.051 (-0.256 – 0.359)	0.735	0.038 (-0.414 – 0.489)	0.866
**Attention**	-0.064 (-0.267 – 0.140)	0.526	0.006 (-0.175 – 0.187)	0.947	-0.254 (-0.770 – 0.262)	0.323	-0.020 (-0.223 – 0.183)	0.843	-0.006 (-0.283 – 0.271)	0.966
**Visual learning**	-0.160 (-0.506 – 0.188)	0.355	-0.380 (-0.704 - 0.057)	0.023	-0.397 (-1.303 – 0.510)	0.378	-0.043 (-0.397 – 0.311)	0.805	-0.387 (-0.891 – 0.117)	0.128
**Executive function N = 32**	-0.021 (-0.230 – 0.188)	0.839	-0.026 (-0.238 – 0.187)	0.805	0.346 (-0.190 – 0.881)	0.197	-0.021 (-0.236 – 0.194)	0.844	-0.060 (-0.370 – 0.250)	0.693
**General cognitive ability**	0.008 (-0.314 – 0.330)	0.961	-0.247 (-0.553 – 0.059)	0.110	-0.295 (-1.124 – 0.534)	0.472	0.007 (-0.317 – 0.331)	0.965	-0.096 (-0.572 – 0.380)	0.683

Supplementary Table 8. Exploratory analyses of interaction effect continued, partial r values for FEP and HC separately

	**Region of interest**																			
	**DLPFC**				**Hippocampus**				**Thalamus**				**ACC**				**Temporal cortex**			
	FEP		HC		FEP		HC		FEP		HC		FEP		HC		FEP		HC	
**Cognitive domain**	Partial *r*	p-value	Partial *r*	p-value	Partial *r*	p-value	Partial *r*	p-value	Partial *r*	p-value	Partial *r*	p-value	Partial *r*	p-value	Partial *r*	p-value	Partial *r*	p-value	Partial *r*	p-value
**Verbal learning**	-	-	-	-	-	-	-	-	-0.293	0.254	0.288	0.297	-0.062	0.814	0.424	0.116	0.229	0.377	0.442	0.099
**Working memory**	-	-	-	-	0.177	0.497	0.049	0.864	0.135	0.607	-0.066	0.816	0.205	0.430	-0.291	0.292	0.038	0.886	-0.229	0.412
**Speed of processing**	-	-	-	-	0.053	0.841	0.270	0.330	0.095	0.717	-0.031	0.912	0.149	0.568	-0.055	0.847	0.026	0.922	-0.065	0.819
**Attention**	0.221	0.394	0.321	0.243	0.539	0.026	0.489	0.064	0.167	0.522	0.542	0.037	0.317	0.216	0.340	0.216	0.492	0.045	0.446	0.096
**Visual learning**	0.014	0.959	0.260	0.349	-0.184	0.479	0.613	0.015	-0.099	0.706	0.249	0.371	0.210	0.419	0.144	0.608	-0.126	0.631	0.402	0.138
**Executive function**	0.224	0.421	0.363	0.183	-0.066	0.815	0.005	0.987	0.371	0.174	-0.050	0.860	0.146	0.603	0.260	0.350	0.144	0.609	0.356	0.194
**General cognitive ability**	0.060	0.819	0.017	0.952	-0.037	0.890	0.499	0.058	-0.072	0.783	0.204	0.466	0.158	0.544	0.072	0.799	0.024	0.928	0.142	0.613

Supplementary Table 9. Exploratory analyses of baseline D2-R levels and cognitive function at 1.5 year follow-up. Only FEP, N = 12

	**Region of interest**														
	**DLPFC**			**Hippocampus**			**Thalamus**			**ACC**			**Temporal cortex**		
**Cognitive domain**	β (95% CI)	Partial *r*	p-value	β (95% CI)	Partial *r*	p-value	β (95% CI)	Partial *r*	p-value	β (95% CI)	Partial *r*	p-value	β (95% CI)	Partial *r*	p-value
**Verbal learning**	-0.114 (-0.334 – 0.107)	-0.362	0.274	0.157 (-0.197 – 0.510)	0.317	0.342	0.139 (0.421 – 5.502)	0.114	0.739	0.048 (-0.266 – 0.361)	0.114	0.740	-0.167 (-0.519 – 0.185)	-0.336	0.312
**Working memory**	-0.049 (-0.162 – 0.064)	-0.310	0.354	0.066 (-0.114 – 0.246)	0.266	0.429	-0.104 (-0.556 – 0.350)	-0.170	0.618	-0.031 (-0.187 – 0.126)	-0.146	0.669	-0.048 (-0.232 – 0.136)	-0.192	0.572
**Speed of processing**	-0.018 (-0.172 – 0.136)	-0.090	0.793	0.069 (-0.169 – 0.307)	0.214	0.528	0.003 (-0.596 – 0.602)	0.004	0.991	0.006 (-0.200 – 0.213)	0.022	0.948	0.017 (-0.228 – 0.261)	0.051	0.882
**Attention**	-0.003 (-0.144 – 0.137)	-0.018	0.959	0.179 (0.003 – 0.354)	0.608	0.047	0.163 (-0.367 – 0.694)	0.226	0.504	0.034 (-0.152 – 0.220)	0.137	0.689	0.071 (-0.145 – 0.286)	0.240	0.477
**Visual learning**	0.037 (-0.115 – 0.188)	0.180	0.597	0.080 (-0.115 – 0.315)	0.248	0.463	0.178 (-0.403 – 0.760)	0.225	0.505	0.029 (-0.176 – 0.233)	0.105	0.760	0.050 (-0.190 – 0.290)	0.155	0.650
**General cognitive ability**	-0.036 (-0.191 – 0.119)	-0.172	0.613	0.097 (-0.140 – 0.335)	0.295	0.378	0.024 (-0.587 – 0.634)	0.029	0.933	0.002 (-0.209 – 0.213)	0.007	0.984	-0.017 (-0.265 – 0.232)	-0.050	0.884

Supplementary Table 10. Exploratory analyses of baseline D2-R levels and change in cognitive function from baseline to 1.5 year follow-up. Only FEP, N = 12

	**Region of interest**														
	**DLPFC**			**Hippocampus**			**Thalamus**			**ACC**			**Temporal cortex**		
**Cognitive domain**	β (95% CI)	Partial *r*	p-value	β (95% CI)	Partial *r*	p-value	β (95% CI)	Partial *r*	p-value	β (95% CI)	Partial *r*	p-value	β (95% CI)	Partial *r*	p-value
**Verbal learning**	-0.021 (-0.161 – 0.120)	-0.333	0.747	0.100 (-0.117 – 0.311)	0.323	0.333	0.269 (-0.246 – 0.784)	0.366	0.268	0.096 (-0.081 – 0.272)	0.379	0.251	-0.064 (-0.282 – 0.154)	-0.216	0.523
**Working memory**	-0.034 (-0.198 – 0.131)	-0.154	0.652	0.028 (-0.238 – 0.293)	0.078	0.820	-0.463 (-1.012 – 0.086)	-0.536	0.089	-0.162 (-0.350 – 0.026)	-0.544	0.084	-0.026 (-0.288 – 0.237)	-0.073	0.830
**Speed of processing**	0.019 (-0.309 – 0.348)	0.044	0.897	0.172 (-0.338 – 0.681)	0.246	0.466	-0.017 (-1.302 – 1.269)	-0.010	0.977	0.022 (-0.420 – 0.465)	0.038	0.912	0.055 (-0.463 – 0.573)	0.080	0.817
**Attention**	0.132 (-0.126 – 0.389)	0.036	0.277	0.303 (-0.074 – 0.679)	0.518	0.103	0.763 (-0.149 – 1.675)	0.534	0.091	0.214 (-0.121 – 0.549)	0.434	0.182	0.188 (-0.225 – 0.600)	0.324	0.330
**Visual learning**	0.038 (-0.069 – 0.144)	0.258	0.444	0.073 (-0.094 – 0.239)	0.312	0.351	0.034 (-0.394 – 0.463)	0.060	0.860	-0.020 (-0.167 – 0.127)	-0.103	0.764	0.060 (-0.107 – 0.228)	0.262	0.436
**General cognitive ability**	0.029 (-0.249 – 0.306)	0.077	0.821	0.323 (-0.049 – 0.694)	0.548	0.081	0.075 (-1.011 – 1.160)	0.052	0.879	-0.036 (-0.410 – 0.338)	-0.072	0.832	0.063 (-0.374 – 0.499)	0.107	0.754

Supplementary Methods:

The WCST was not used in any of the longitudinal analyses, due to significant learning effects.

Supplementary Table 11. Demographic and clinical data of [^{11}C]FLB 457 patients who completed cognitive testing at follow-up

	**FEP N=12**
**Gender (male/female)**	8/4
**Age**	30.3 (7.7)
**Education in years**	14.9 (4.4) N = 10
**Diagnosis**	
**Schizophrenia**	4
**Psychotic disorder NOS**	3
**No diagnosis**	1
**Delusional disorder**	2
**Schizoaffective disorder**	1
**Depressive episode**	1
**PANSS**	
**Positive**	11.8 (4.1)
**Negative**	10.2 (3.2)
**General**	27.6 (8.9)
**Total**	49.6 (13.1)
**Level of functioning**	
**CGI**	2.5 (1.5)
**GAF**	62.1 (20.3)
**Medication at time of testing**	
**Anti-psychotics**	4 N = 10
**Anti-depressants**	0 N = 9
**Mood stabilizers**	0 N = 10
**Benzodiazepines**	2 N = 6

Detailed information on age correction of test scores:
Cognitive test scores were age-corrected using linear regression (Raw Cognitive Test Score ~ Age) using the full study sample (see Supplementary Table 2 for demographics). The resulting residuals were used as the cognitive variables in all analyses. The match between raw scores and age-corrected residuals were plotted and inspected visually. Cognitive composite measures, (Speed of Processing (SoP), Working Memory (WM) and Neurocognitive composite (NC)) were constructed as described in the MCCB manual (Nuechterlein & Green, 2006) by adding together the relevant tests and dividing this by the number of tests. One of the test scores included in the SoP domain (TMT) was flipped, so that higher values represented better performance. Before analysis, all cognitive measures were standardized into z-values, with a mean of 0 and a standard deviation of 1.

Supplementary Table 12, demographic data on larger cohort at baseline, used to construct age corrected variables.

	**FEP N=83**	**HC N=59**
**Gender (male/female)**	50/33	28/31
**Age**	28.9 (7.9)	27.1 (5.6)
**Education in years**	14.0 (3.1) N=72	14.9 (2.2) N = 58
**Duration of illness (months**	11.4 (17.2) N = 69	-
**Diagnosis**		-
**Schizophrenia**	18	
**Schizophreniform disorder**	26	
**Psychotic disorder NOS**	28	
**Delusional disorder**	5	
**Schizoaffective disorder**	2	
**Brief psychotic disorder**	3	
**Severe depressive disorder with psychotic features**	1	
**PANSS**		-
**Positive**	18.2 (5.8)	
**Negative**	16.6 (6.7)	
**General**	36.5 (10.7)	
**Total**	71.3 (19.6)	
**Level of functioning**		-
**CGI**	4.4 (1.2)	
**GAF**	42.2 (13.1) N = 82	
**Medication at time of testing**		-
**Anti-psychotics**	43	
**Anti-depressants**	8	
**Mood stabilizers**	2	
**Benzodiazepines**	43	

Supplementary Table 13, demographic data on larger cohort at 1,5 year follow-up, used to construct age corrected variables.

	FEP N=37	HC N=25
**Gender (male/female)**	21/16	11/14
**Age**	32.4 (9.2)	28.2 (6.3)
**Education in years**	14.1 (3.2) N = 35	16.2 (2.6)
**Diagnosis**		-
**Schizophrenia**	21	
**Psychotic disorder NOS**	5	
**No diagnosis**	3	
**Delusional disorder**	2	
**Schizoaffective disorder**	2	
**Depressive episode**	2	
**Brief psychotic disorder**	1	
**Residual Schizophrenia**	1	
**PANSS**		-
**Positive**	11.4 (3.7)	
**Negative**	13.8 (5.8)	
**General**	27.4 (7.5)	
**Total**	52.6 (13.8)	
**Level of functioning**		-
**CGI**	2.7 (1.4) N = 36	
**GAF**	64.2 (18.1) N = 36	
**Medication at time of testing**		-
**Anti-psychotics**	15 N = 31	
**Anti-depressants**	7 N = 30	
**Mood stabilizers**	2 N = 35	
**Benzodiazepines**	3 N = 24	
