# Supplementary material for: No association between cortical dopamine D2 receptor availability and cognition in antipsychotic-naive first-episode psychosis
Source: NPJ Schizophr. 2021 Sep 21;7:46. doi: 10.1038/s41537-021-00176-x (PMC8455597; doi:10.1038/s41537-021-00176-x)
Supplement: Supplementary file 2 — Reporting Summary [file 41537_2021_176_MOESM2_ESM.pdf]

nature research

Corresponding author(s): Maria Lee

Last updated by author(s): Apr 12, 2021

Reporting Summary

Nature Research wishes to improve the reproducibility of the work that we publish. This form provides structure for consistency and transparency in reporting. For further information on Nature Research policies, see our Editorial Policies and the Editorial Policy Checklist.

Statistics

For all statistical analyses, confirm that the following items are present in the figure legend, table legend, main text, or Methods section.

n/a	Confirmed	
☐	☒	The exact sample size (*n*) for each experimental group/condition, given as a discrete number and unit of measurement
☐	☒	A statement on whether measurements were taken from distinct samples or whether the same sample was measured repeatedly
☐	☒	The statistical test(s) used AND whether they are one- or two-sided<br>*Only common tests should be described solely by name; describe more complex techniques in the Methods section.*
☐	☒	A description of all covariates tested
☐	☒	A description of any assumptions or corrections, such as tests of normality and adjustment for multiple comparisons
☐	☒	A full description of the statistical parameters including central tendency (e.g. means) or other basic estimates (e.g. regression coefficient) AND variation (e.g. standard deviation) or associated estimates of uncertainty (e.g. confidence intervals)
☐	☒	For null hypothesis testing, the test statistic (e.g. *F*, *t*, *r*) with confidence intervals, effect sizes, degrees of freedom and *P* value noted<br>*Give P values as exact values whenever suitable.*
☐	☒	For Bayesian analysis, information on the choice of priors and Markov chain Monte Carlo settings
☒	☐	For hierarchical and complex designs, identification of the appropriate level for tests and full reporting of outcomes
☐	☒	Estimates of effect sizes (e.g. Cohen's *d*, Pearson's *r*), indicating how they were calculated

Our web collection on statistics for biologists contains articles on many of the points above.

Software and code

Policy information about availability of computer code

Data collection	No software was used in data collection
Data analysis	The code used to analyze data and produce figures is publicly available at https://github.com/MariaLeeR/D2_cognition_FEP

For manuscripts utilizing custom algorithms or software that are central to the research but not yet described in published literature, software must be made available to editors and reviewers. We strongly encourage code deposition in a community repository (e.g. GitHub). See the Nature Research guidelines for submitting code & software for further information.

Data

Policy information about availability of data

All manuscripts must include a data availability statement. This statement should provide the following information, where applicable:

- Accession codes, unique identifiers, or web links for publicly available datasets
- A list of figures that have associated raw data
- A description of any restrictions on data availability

Due to institutional restrictions, the data cannot be shared openly but can instead be made available upon request on a case by case basis as allowed by the legislation and ethical permits. Requests for access can be made to the Karolinska Institutet's Research Data Office at rdo@ki.se.

Field-specific reporting

Please select the one below that is the best fit for your research. If you are not sure, read the appropriate sections before making your selection.

☒ Life sciences ☐ Behavioural & social sciences ☐ Ecological, evolutionary & environmental sciences

For a reference copy of the document with all sections, see nature.com/documents/nr-reporting-summary-flat.pdf

Life sciences study design

All studies must disclose on these points even when the disclosure is negative.

Sample size	No sample size calculation was performed. Instead sample size was determined based on previous literature, with studies using a similar sample size (N ~ 20 per group) being able to demonstrate group differences as well as associations between biomarkers and symptom levels.
Data exclusions	20 first episode psychosis patients were recruited for the study. One was later excluded due to brain abnormalaties demonstrated by MRI scan and age (65 year at scan). One additional patient was excluded due to missing MRI.
Replication	The code for the analysis has been quality controlled and executed several times. All attempts at replication of the results were succesful.
Randomization	Allocation was not random, participants were grouped depending on whether they were first-episode psychosis patients or healthy controls. To adjust for covariates, healthy controls were matched to patients on gender and age (+/- 2 years)
Blinding	Blinding was not possible, due to the nature of the condition studied. Psychosis is oftentimes apparent to the observer, and even when it is not, those with psychosis require more support and careful treatment than healthy controls during study procedures to not cause undue stress.

Reporting for specific materials, systems and methods

We require information from authors about some types of materials, experimental systems and methods used in many studies. Here, indicate whether each material, system or method listed is relevant to your study. If you are not sure if a list item applies to your research, read the appropriate section before selecting a response.

Materials & experimental systems

n/a	Involved in the study	
☒	☐	Antibodies
☒	☐	Eukaryotic cell lines
☒	☐	Palaeontology and archaeology
☒	☐	Animals and other organisms
☐	☒	Human research participants
☒	☐	Clinical data
☒	☐	Dual use research of concern

Methods

n/a	Involved in the study	
☒	☐	ChIP-seq
☒	☐	Flow cytometry
☒	☐	MRI-based neuroimaging

Human research participants

Policy information about studies involving human research participants

Population characteristics	First episode psychosis patients, N = 18. Gender: 11 male, 7 female. Mean age 28.9 (standard deviation 6.3 years), range: 18 - 42). Diagnosis: Schizophrenia (N = 6), schizophreniform disorder (N=5), psychotic disorder NOS (N=4) or delusional disorder (N=3) according to DSM-IV. At time of PET, none of the first episode psychosis patients were receiving anti-psychotic medication. At time of cognitive testing, two patients had been prescribed first generation anti-psychotic medication with 3 and 6 days of total exposure respectively.<br>Healthy control subjects, N = 16. Gender: 10 male, 6 female. Mean age 29.3 (standard deviation 6.2 years), range 20-43. All healthy control subjects were healthy and had no history of psychiatric illness or treatment.
Recruitment	First episode psychosis patients were recruited through psychiatric clinics in Stockholm, both in-patient wards and out patient clinics. Given that patients were required to be naive to anti-psychotic drugs and be able to provide informed consent, selection bias must be considered. Our sample do not represent all first-episode psychosis patients, as the most severely ill and confused would not have been brought to our attention. Other inclusion criteria that limit the generalizability of our findings is that we required patients to be able to speak and understand Swedish or English, and that those with current drug use or a previous diagnosis of drug abuse were excluded. Our findings must be interpreted in the light of this selection process.<br>Healthy control subjects were recruited through advertisement, with the inherent selection bias this introduces. They were age and gender matched to the first episode psychosis patients. In the present study, we also corrected for any residual age effects on cognition. As our healthy controls might be more healthy and research interested than the general population, this could have inflated any potential group differences. However, in this study first episode psychosis patients and healthy

control subjects were grouped together in the regression analysis, and we did not look directly at group differences even if we did inspect the groups separately.

Ethics oversight	Regional Ethics Committee in Stockholm and the Radiation Safety Committee of the Karolinska University Hospital approved the protocol (diary number: 2010/879-31-1)

Note that full information on the approval of the study protocol must also be provided in the manuscript.
